# Supplementary material for: Atomic-resolution structures from polycrystalline covalent organic frameworks with enhanced cryo-cRED
Source: Nat Commun. 2022 Jul 11;13:4016. doi: 10.1038/s41467-022-31524-9 (PMC9276740; doi:10.1038/s41467-022-31524-9)
Supplement: Supplementary file 1 — Supplementary Information [file 41467_2022_31524_MOESM1_ESM.pdf]

Supplementary Information

Atomic-resolution structures from polycrystalline covalent organic frameworks with enhanced cryo-cRED

Jian Li[1,2,#], Cong Lin[1,3], Tianqiong Ma[1], Junliang Sun[1,*]

1 College of Chemistry and Molecular Engineering, Beijing National Laboratory for Molecular Sciences, Peking University, 100871 Beijing, China.

2 Berzelii Center EXSELENT on Porous Materials, Department of Materials and Environmental Chemistry, Stockholm University, 10691 Stockholm, Sweden.

3 Department of Mechanical Engineering, The Hong Kong Polytechnic University, 999077 Hong Kong, China.

* Corresponding author emails: junliang.sun@pku.edu.cn

# Present address: Department of Fibre and Polymer Technology, School of Engineering Sciences in Chemistry, Biotechnology and Health, KTH Royal Institute of Technology, Tekninkringen 56-58, Stockholm, SE-100 44, Sweden.

Table of Contents

Section 1: Supplementary figures and tables

Supplementary Fig. 1 Schematic of the synthesis of E-FCOF-5 and C-FCOF-5 (a), 3D-TPB-COF-OMe, 3D-TPB-COF-Me and 3D-TPB-COF-OH (b).

Supplementary Fig. 2 Powder X-ray diffraction (PXRD) pattern of E-FCOF-5 and C-FCOF-5 (a), 3D-TPB-COF-OMe, 3D-TPB-COF-Me and 3D-TPB-COF-OH (b).

Supplementary Fig. 3 Scanning electron microscope (SEM) image of FCOF-5 (a), 3D-TPB-COF-OMe (b), 3D-TPB-COF-Me (c) and 3D-TPB-COF-OH (d).

Supplementary Fig. 4 (a) Overview of the E-FCOF-5 nano-crystals prepared on the TEM grid; (b) ED pattern of E-FCOF-5 with a high resolution of 0.82 Å at the beginning of the cRED data collection; (c) ED pattern of E-FCOF-5 at the final stage of cRED data collection.

Supplementary Fig. 5 Reconstructed 3D reciprocal lattice of E-FCOF-5. (a) Overview of reconstructed lattice; (*h*0*l*) (b), (0*kl*) (c), and (*hk*0) (d) slices cut from the reconstructed reciprocal lattice.

Supplementary Fig. 6 Reconstructed 3D reciprocal lattice from the 22 cRED datasets of E-FCOF-5. The 16 datasets marked with # belong to the selected cluster for structure solution and refinement.

Supplementary Fig. 7 Six-fold interpenetrated pts topology of E-FCOF-5 (left), and C-FCOF-5 (right).

Supplementary Fig. 8 Reconstructed 3D reciprocal lattice from the 22 cRED datasets of C-FCOF-5. The 16 datasets marked with # belong to the selected cluster for structure solution and refinement.

Supplementary Fig. 9 Reconstructed 3D reciprocal lattice of C-FCOF-5. (a) Overview of the reconstructed lattice; (*h*0*l*) (b), (0*kl*) (c), and (*hk*0) (d) slices cut from the reconstructed reciprocal lattice.

Supplementary Fig. 10 ED pattern of C-FCOF-5 with a high resolution of 0.79 Å during cRED data collection.

Supplementary Fig. 11 The geometry of TFMB in E-FCOF-5 (a) (e, g, pink) and C-FCOF (b)(f, g, orange) obtained by refinement of cryo-cRED data at atomic resolution (this study). The geometry of TFMB in E-FCOF-5 (c) (e, dark green) and C-FCOF (d)(f, light green) obtained by PXRD simulation (previous study1). (e) The geometry comparison of TFMB between (a) and (c). (f) The geometry comparison of TFMB between (b) and (d). (g) The geometry comparison of TFMB between (a) and (b).

Supplementary Fig. 12 Pattern of 3D-TPB-COF-OMe with a high resolution of 0.87 Å during cRED data collection

Supplementary Fig. 13 Reconstructed 3D reciprocal lattice of 3D-TPB-COF-OMe. (a) Overview of the reconstructed lattice; (*h*0*l*) (b), (0*kl*) (c), and (*hk*0) (d) slices cut from the reconstructed reciprocal lattice.

Supplementary Fig. 14 Potential density maps of 3D-TPB-COF-OMe from refinement against on a single dataset (dataset 4# in table S1) with 65.9% completeness.

Supplementary Fig. 15 ED pattern of 3D-TPB-COF-Me with a high resolution of 0.90Å during cRED data collection.

Supplementary Fig. 16 Reconstructed 3D reciprocal lattice of 3D-TPB-COF-Me. (a) Overview of the reconstructed lattice; (*h*0*l*) (b), (0*kl*) (c), and (*hk*0) (d) slices cut from the reconstructed reciprocal lattice.

Supplementary Fig. 17 ED pattern of 3D-TPB-COF-OH with a resolution of 1.2Å during cRED data collection.

Supplementary Fig. 18 Reconstructed 3D reciprocal lattice of 3D-TPB-COF-OH. (a) Overview of the reconstructed lattice; (*h0l*) (b), (*hk*0) (c), and (0*kl*) (d) slices cut from the reconstructed reciprocal lattice.

Supplementary Fig. 19 (a) Reconstructed 3D reciprocal lattice from the 5 cRED datasets of 3D-TPB-COF-OH. The datasets marked with # belong to the selected cluster for structure solution and refinement. (b) The cut distance in dendrogram is represented by the blue line at 0.41 for HCA, 4 cRED datasets for 3D-TPB-COF-OH (in orange), which were selected for the structure determination and refinement.

Supplementary Fig. 20 The porous structure of 3D-TPB-COF-OMe (a), 3D-TPB-COF-Me (d) and 3D-TPB-COF-OH(g). Single pts network of 3D-TPB-COF-OMe (b), 3D-TPB-COF-Me (e).and 3D-TPB-COF-OH (h). The five-fold interpenetrated pts topology of 3D-TPB-COF-OMe (c), 3D-TPB-COF-Me (f) and 3D-TPB-COF-OH. Color scheme: C, gray; N, blue; H, white.

Supplementary Fig. 21 Ortep representation of 3D COFs with 50% probability ellipsoids. (a) E-FCOF-5 (CCDC number: 2115021), (b) C-FCOF-5 (CCDC number: 2115022), (c) 3D-TPB-COF-OMe (CCDC number: 2115023), (d) 3D-TPB-COF-Me (CCDC number: 2115025), and (e) 3D-TPB-COF-OH (CCDC number: 2115024).

Supplementary Table 1 Summary of the cRED indexing and processing results for the E-FCOF-5 crystals.

Supplementary Table 2 cRED experimental parameters, crystallographic data, and structure refinement details of E-FCOF-5.

Supplementary Table 3 Summary of the cRED datasets indexing and processing results for the C-FCOF-5 crystals.

Supplementary Table 4 cRED experimental parameters, crystallographic data, and structure refinement details of C-FCOF-5.

Supplementary Table 5 Summary of the cRED datasets indexing and processing results for all the 3D-TPB-COF-OMe crystals.

Supplementary Table 6 cRED experimental parameters, crystallographic data, and structure refinement details of 3D-TPB-COF-OMe

Supplementary Table 7 Summary of the cRED datasets indexing and processing results for all the 3D-TPB-COF-Me crystals.

Supplementary Table 8 cRED experimental parameters, crystallographic data, and structure refinement details of 3D-TPB-COF-Me

Supplementary Table 9 Summary of the cRED datasets indexing and processing results for the 3D-TPB-COF-OH crystals.

Supplementary Table 10 cRED experimental parameters, crystallographic data, and structure refinement details of 3D-TPB-COF-OH.

Section 2: References

Section 1: Supplementary figures and tables

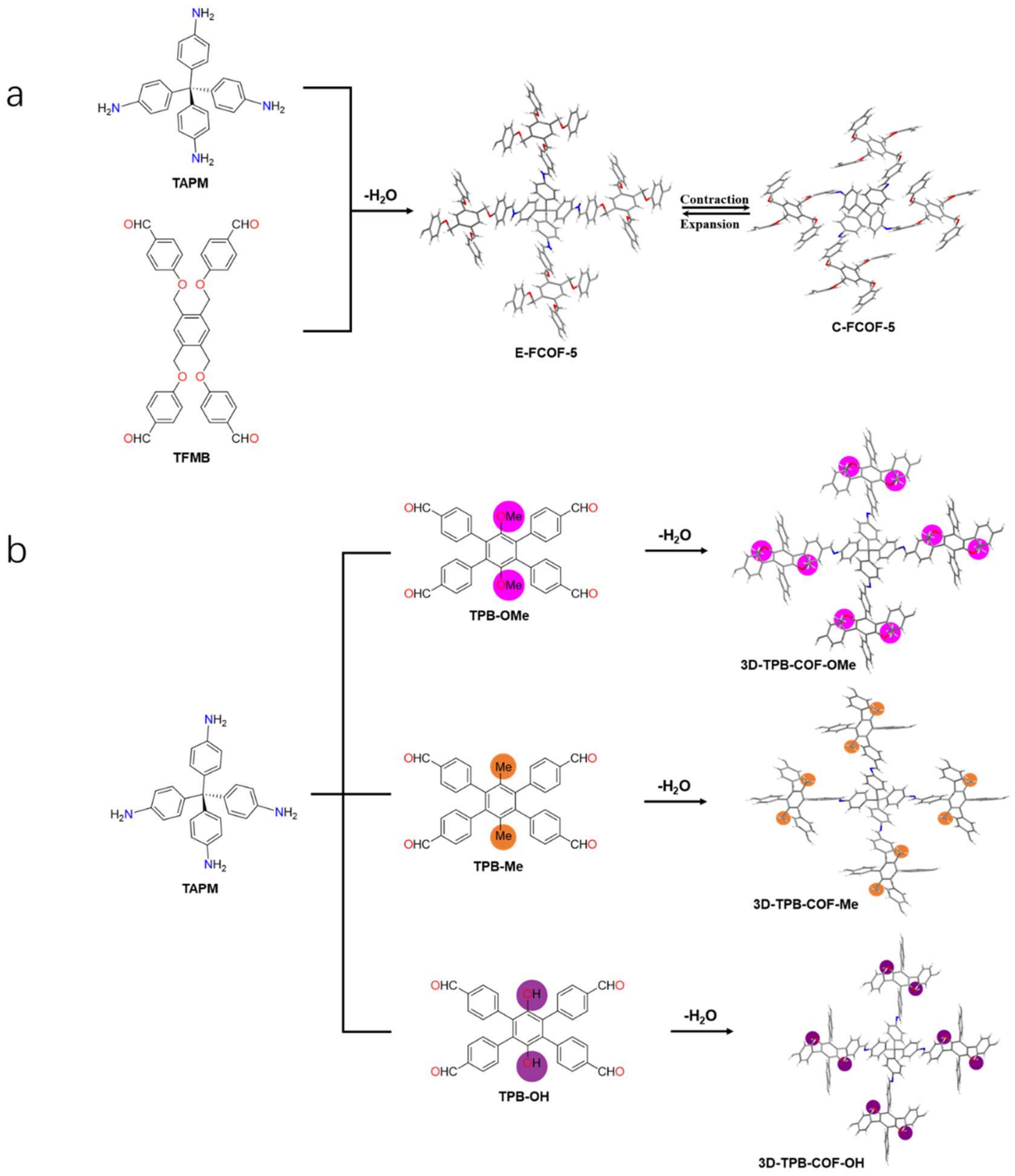

Supplementary Fig. 1 Schematic of the synthesis of E-FCOF-5 and C-FCOF-5 (a), 3D-TPB-COF-OMe, 3D-TPB-COF-Me and 3D-TPB-COF-OH (b).

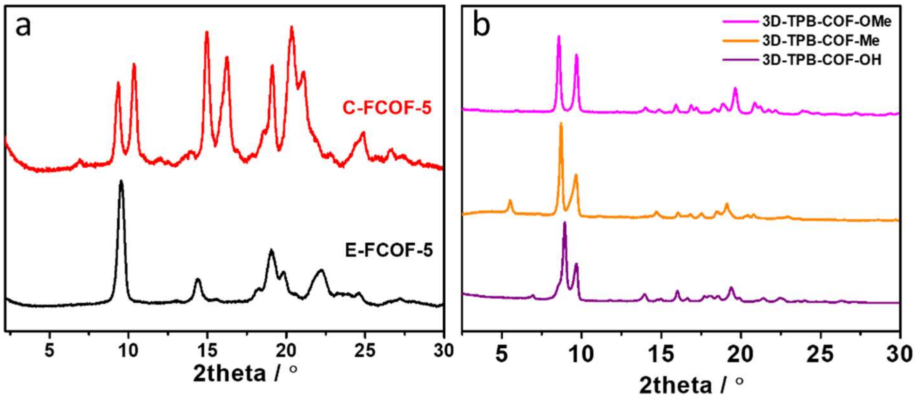

Supplementary Fig. 2 Powder X-ray diffraction **(**PXRD) pattern of E-FCOF-5 and C-FCOF-5 (a), 3D-TPB-COF-Ome, 3D-TPB-COF-Me and 3D-TPB-COF-OH (b).

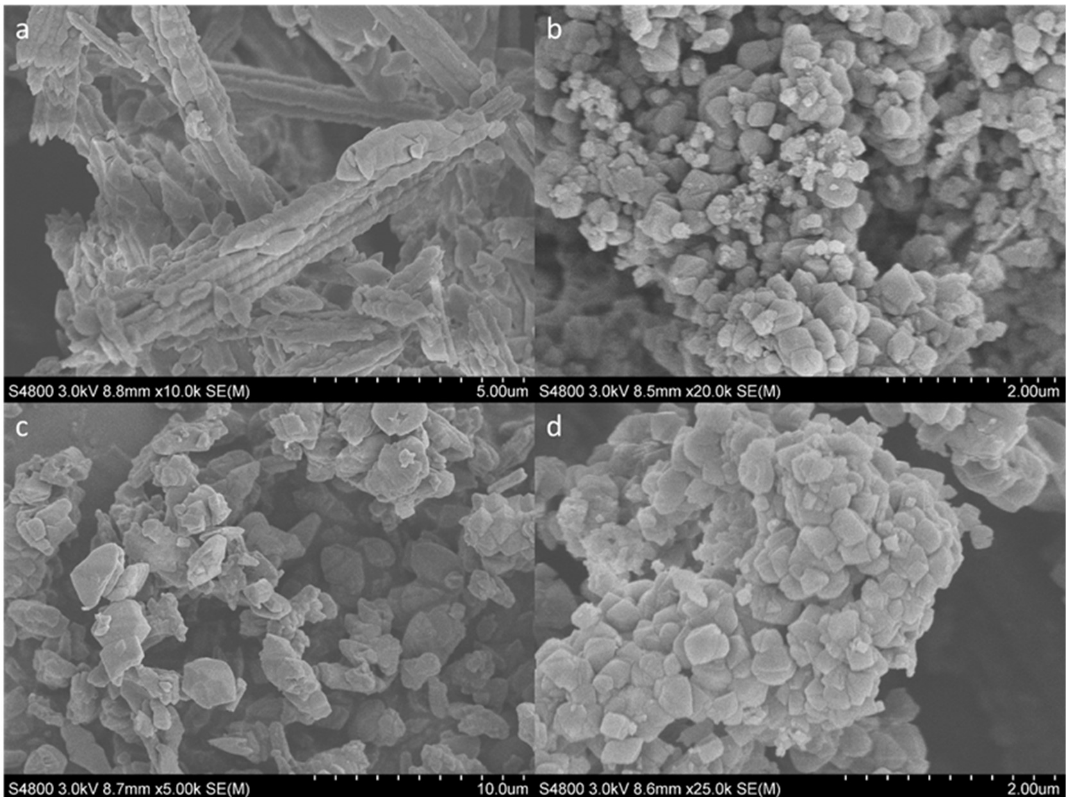

Supplementary Fig. 3 Scanning electron microscope (SEM) image of FCOF-5 (a), 3D-TPB-COF-OMe (b), 3D-TPB-COF-Me (c), and 3D-TPB-COF-OH (d).

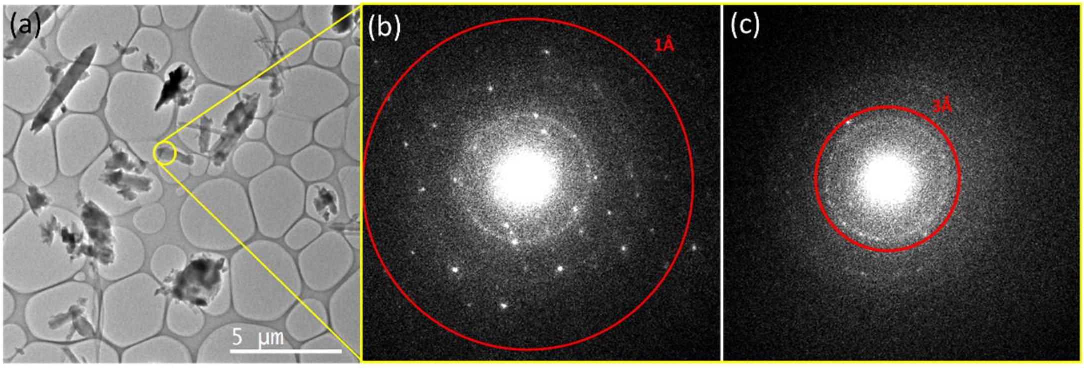

Supplementary Fig. 4 (a) Overview of the E-FCOF-5 nano-crystals prepared on the TEM grid; (b) ED pattern of E-FCOF-5 with a high resolution of 0.82 Å at the beginning of the cRED data collection; (c) ED pattern of E-FCOF-5 at the final stage of cRED data collection.

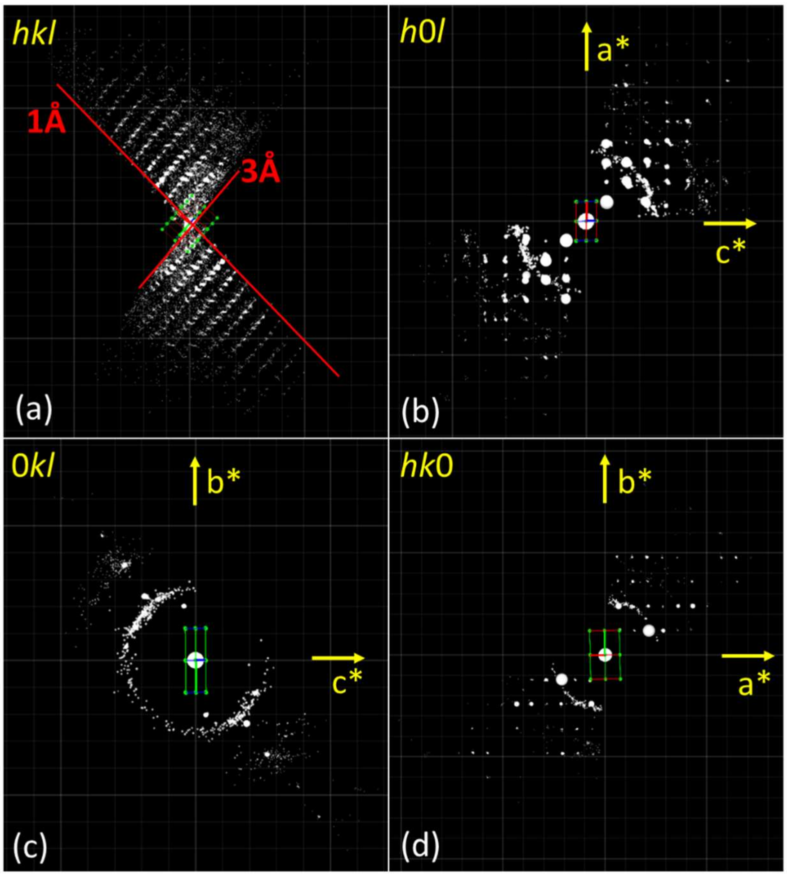

Supplementary Fig. 5 Reconstructed 3D reciprocal lattice of E-FCOF-5. (a) Overview of reconstructed lattice; (*h0l*) (b), (*0kl*) (c), and (*hk*0) (d) slices cut from the reconstructed reciprocal lattice.

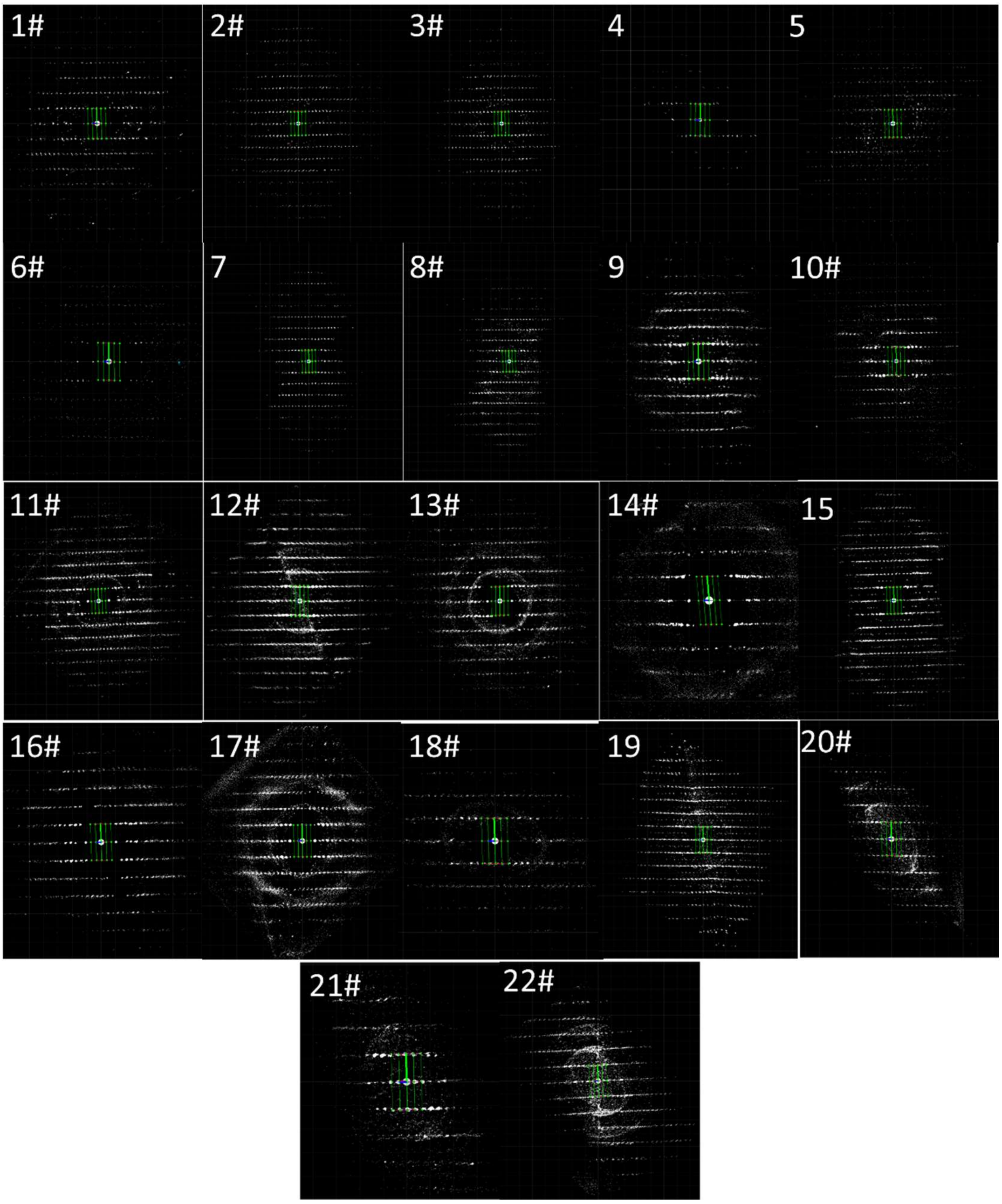

Supplementary Fig. 6 Reconstructed 3D reciprocal lattice from the 22 cRED datasets of E-FCOF-5. The 16 datasets marked with # belong to the selected cluster for structure solution and refinement.

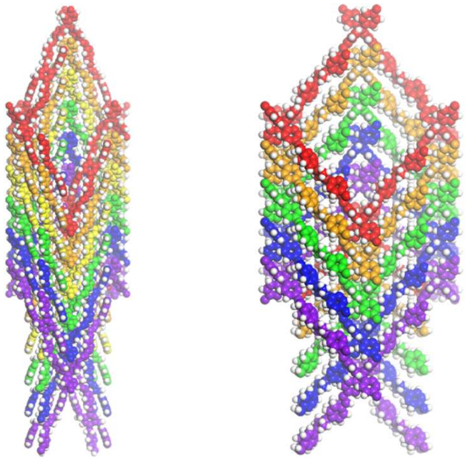

Supplementary Fig. 7 Six-fold interpenetrated **pts** topology of E-FCOF-5 (left) and C-FCOF-5 (right).

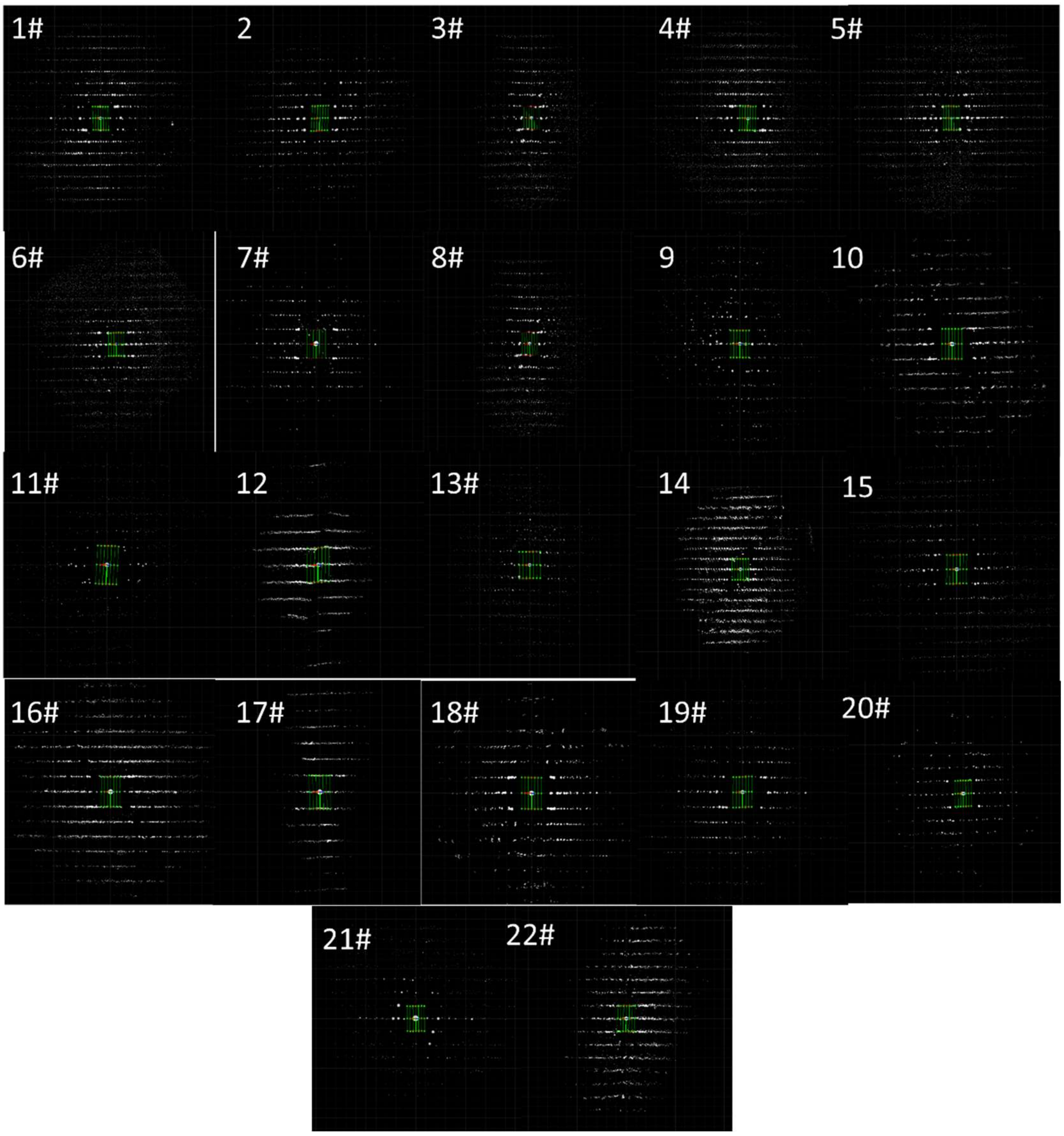

Supplementary Fig. 8 Reconstructed 3D reciprocal lattice from the 22 cRED datasets of C-FCOF-5. The 16 datasets marked with # belong to the selected cluster for structure solution and refinement.

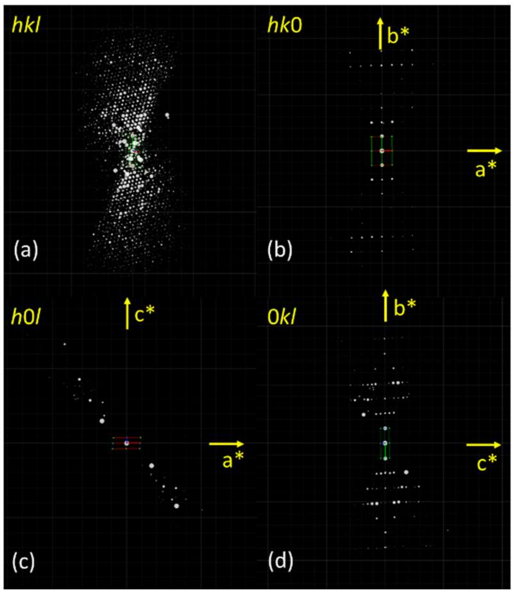

Supplementary Fig. 9 Reconstructed 3D reciprocal lattice of C-FCOF-5. (a) Overview of the reconstructed lattice; (*h0l*) (b), (*0kl*) (c), and (*hk*0) (d) slices cut from the reconstructed reciprocal lattice.

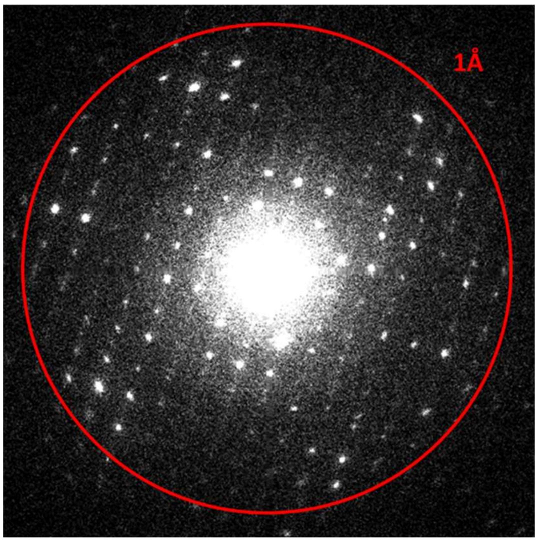

Supplementary Fig. 10 ED pattern of C-FCOF-5 with a high resolution of 0.79Å during cRED data collection.

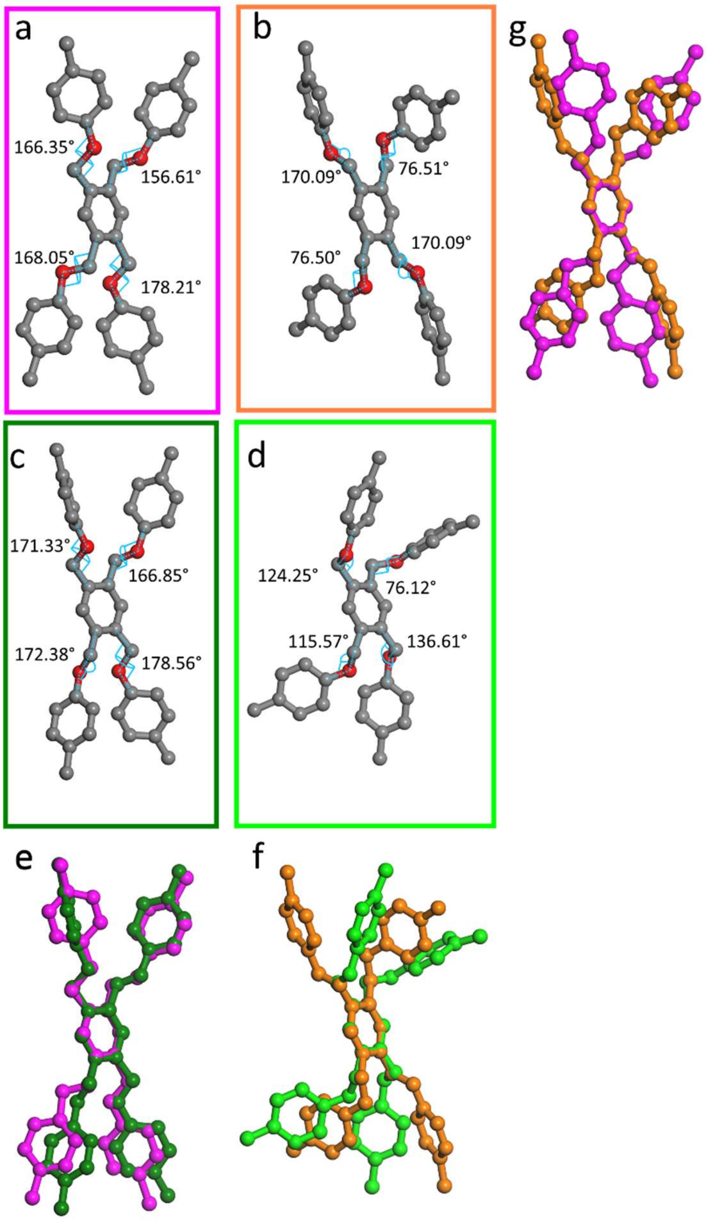

Supplementary Fig. 11 The geometry of TFMB in E-FCOF-5 (a) (e, g, pink) and C-FCOF (b)(f, g, orange) obtained by refinement of cryo-cRED data at atomic resolution (this study). The geometry of TFMB in E-FCOF-5 (c) (e, dark green) and C-FCOF (d)(f, light green) obtained by PXRD simulation (previous study[1]). (e) The geometry comparison of TFMB between (a) and (c). (f) The geometry comparison of TFMB between (b) and (d). (g) The geometry comparison of TFMB between (a) and (b).

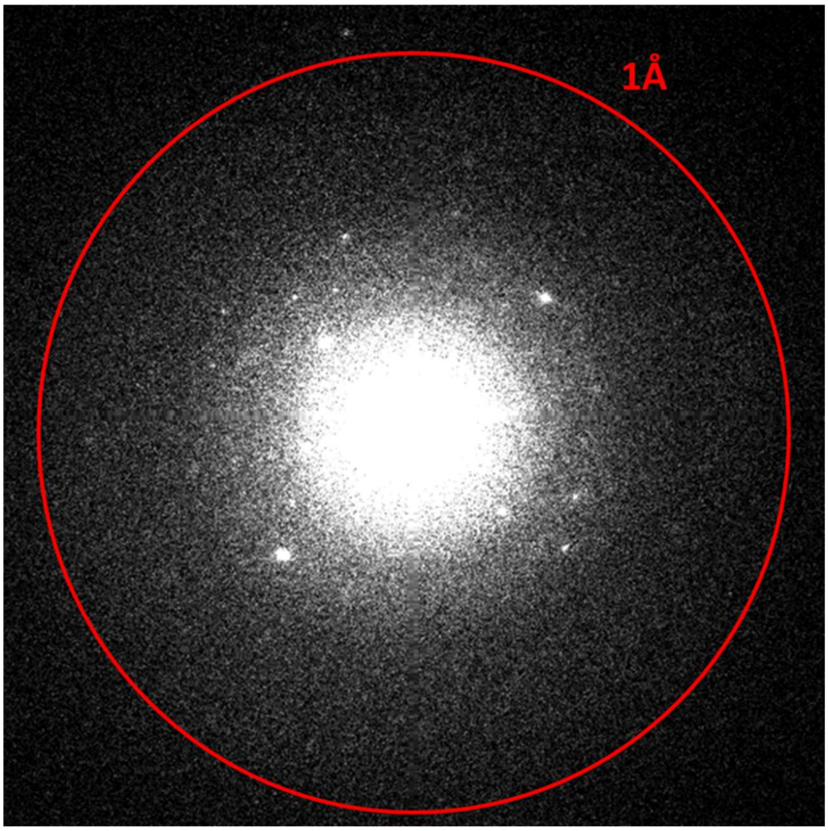

Supplementary Fig. 12 ED pattern of 3D-TPB-COF-OMe with a high resolution of 0.87 Å during cRED data collection

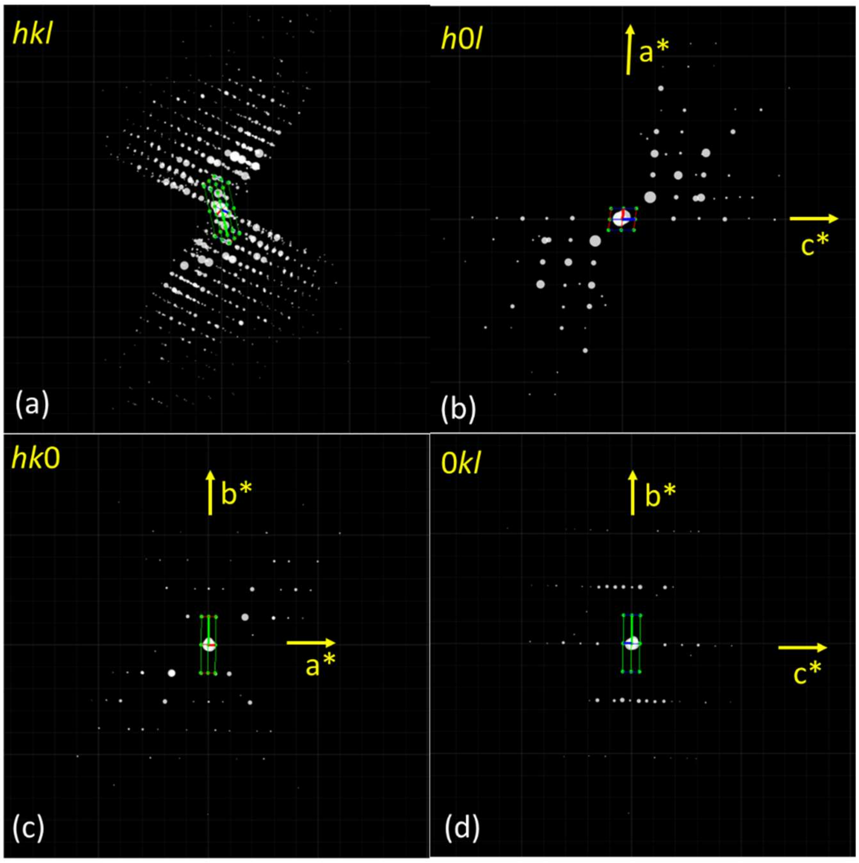

Supplementary Fig. 13 Reconstructed 3D reciprocal lattice of 3D-TPB-COF-OMe. (a) Overview of the reconstructed lattice; (*h0l*) (b), (*0kl*) (c), and (*hk*0) (d) slices cut from the reconstructed reciprocal lattice.

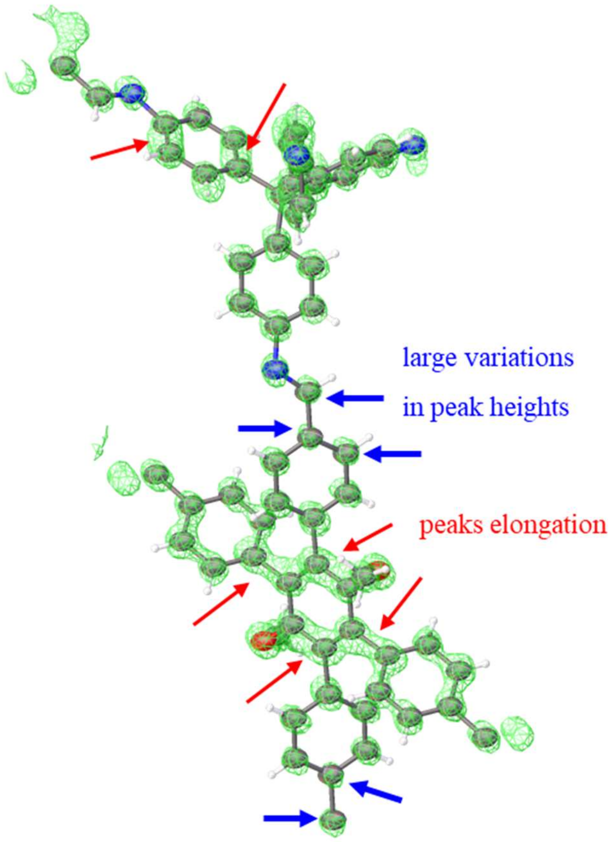

Supplementary Fig. 14 Potential density maps of 3D-TPB-COF-OMe from refinement against on a single dataset (dataset 4# in table S1) with 65.9% completeness.

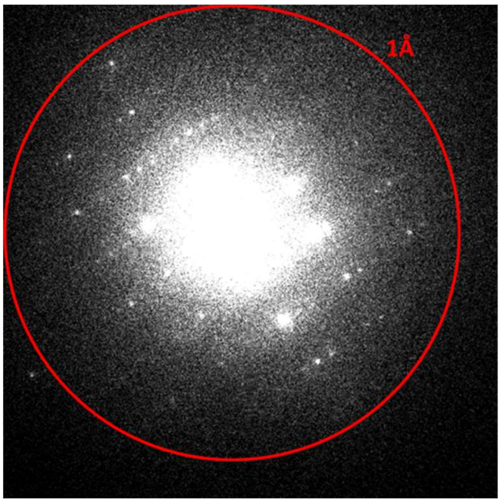

Supplementary Fig. 15 ED pattern of 3D-TPB-COF-Me with a high resolution of 0.90Å during cRED data collection

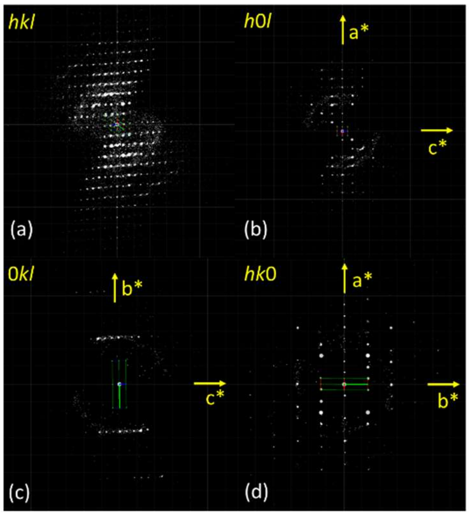

Supplementary Fig. 16 Reconstructed 3D reciprocal lattice of 3D-TPB-COF-Me. (a) Overview of the reconstructed lattice; (*h0l*) (b), (*0kl*) (c), and (*hk*0) (d) slices cut from the reconstructed reciprocal lattice.

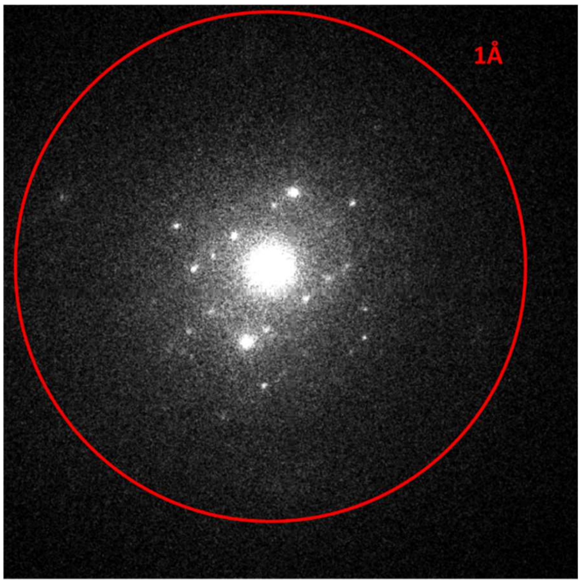

Supplementary Fig. 17 ED pattern of 3D-TPB-COF-OH with a resolution of 1.2Å during cRED data collection.

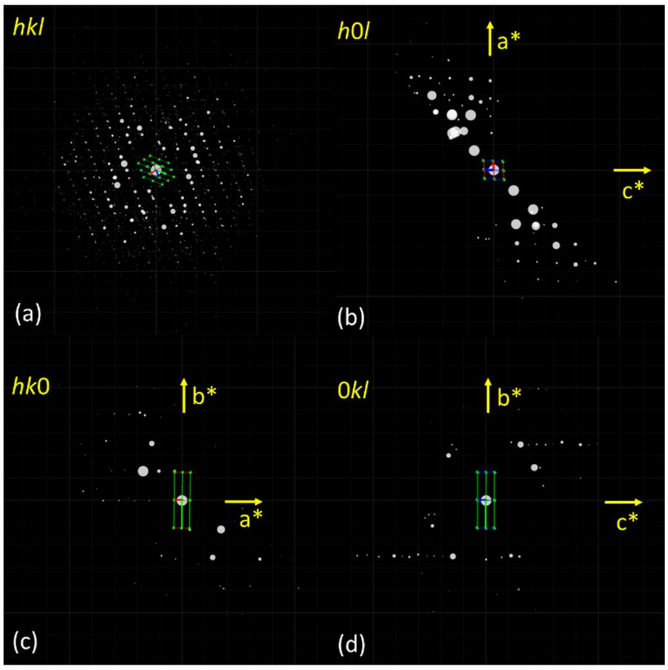

Supplementary Fig. 18 Reconstructed 3D reciprocal lattice of 3D-TPB-COF-OH. (a) Overview of the reconstructed lattice; (*h*0*l*) (b), (*hk*0) (c), and (0*kl*) (d) slices cut from the reconstructed reciprocal lattice.

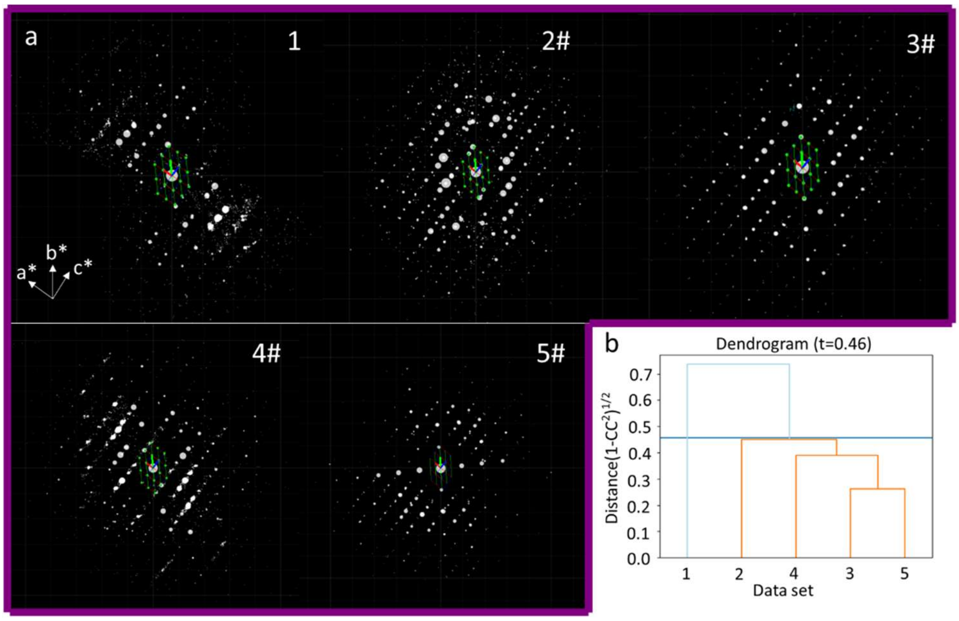

Supplementary Fig. 19 (a) Reconstructed 3D reciprocal lattice from the 5 cRED datasets of 3D-TPB-COF-OH. The datasets marked with # belong to the selected cluster for structure solution and refinement. (b) The cut distance in dendrogram is represented by the blue line at 0.41 for HCA, 4 cRED datasets for 3D-TPB-COF-OH (in orange), which were selected for the structure determination and refinement.

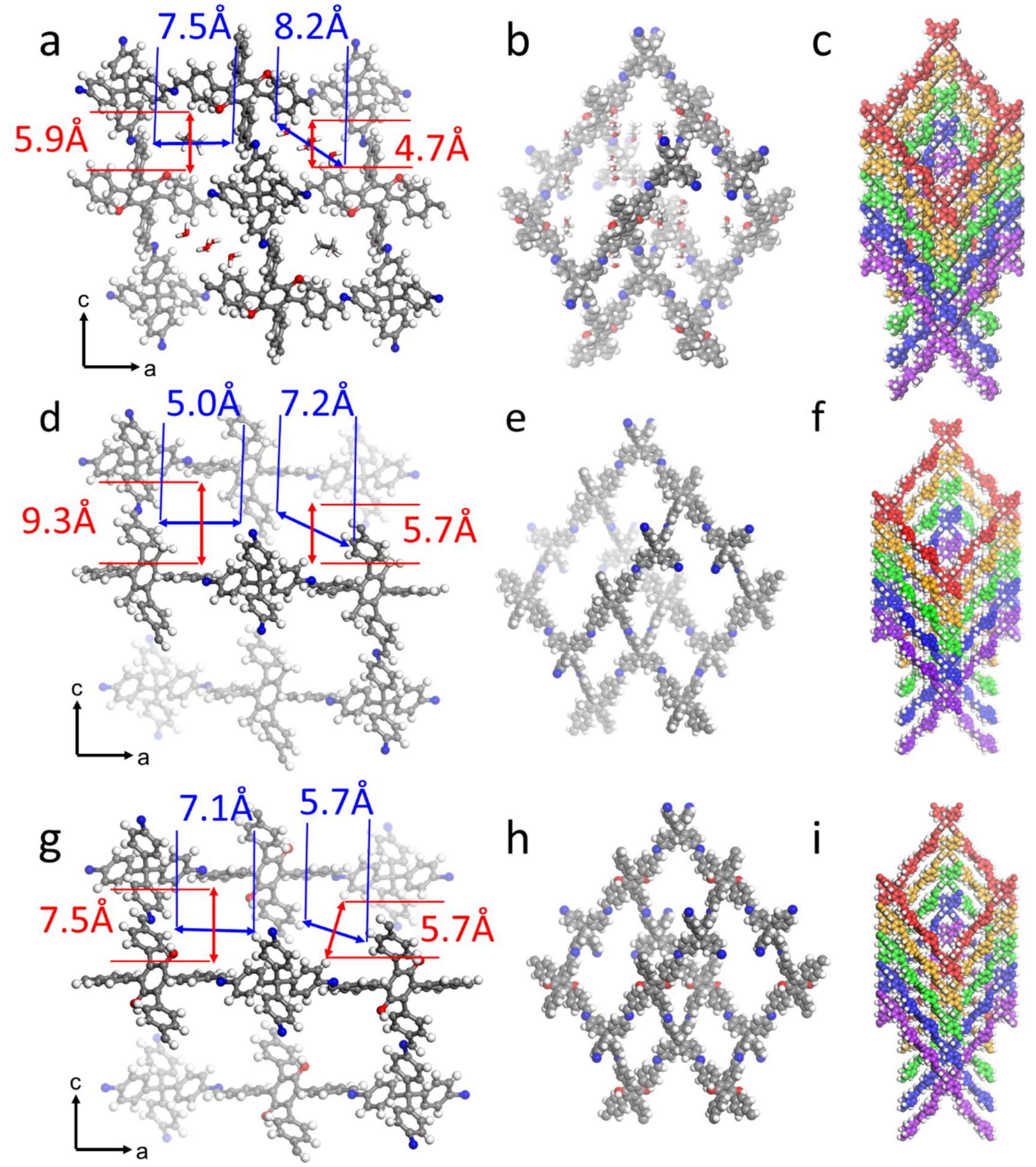

Supplementary Fig. 20 The porous structure of 3D-TPB-COF-OMe (a), 3D-TPB-COF-Me (d) and 3D-TPB-COF-OH (g). Single pts network of 3D-TPB-COF-OMe (b), 3D-TPB-COF-Me (e).and 3D-TPB-COF-OH (h). The five-fold interpenetrated **pts** topology of 3D-TPB-COF-OMe (c), 3D-TPB-COF-Me (f) and 3D-TPB-COF-OH (i). Color scheme: C, gray; N, blue; H, white.

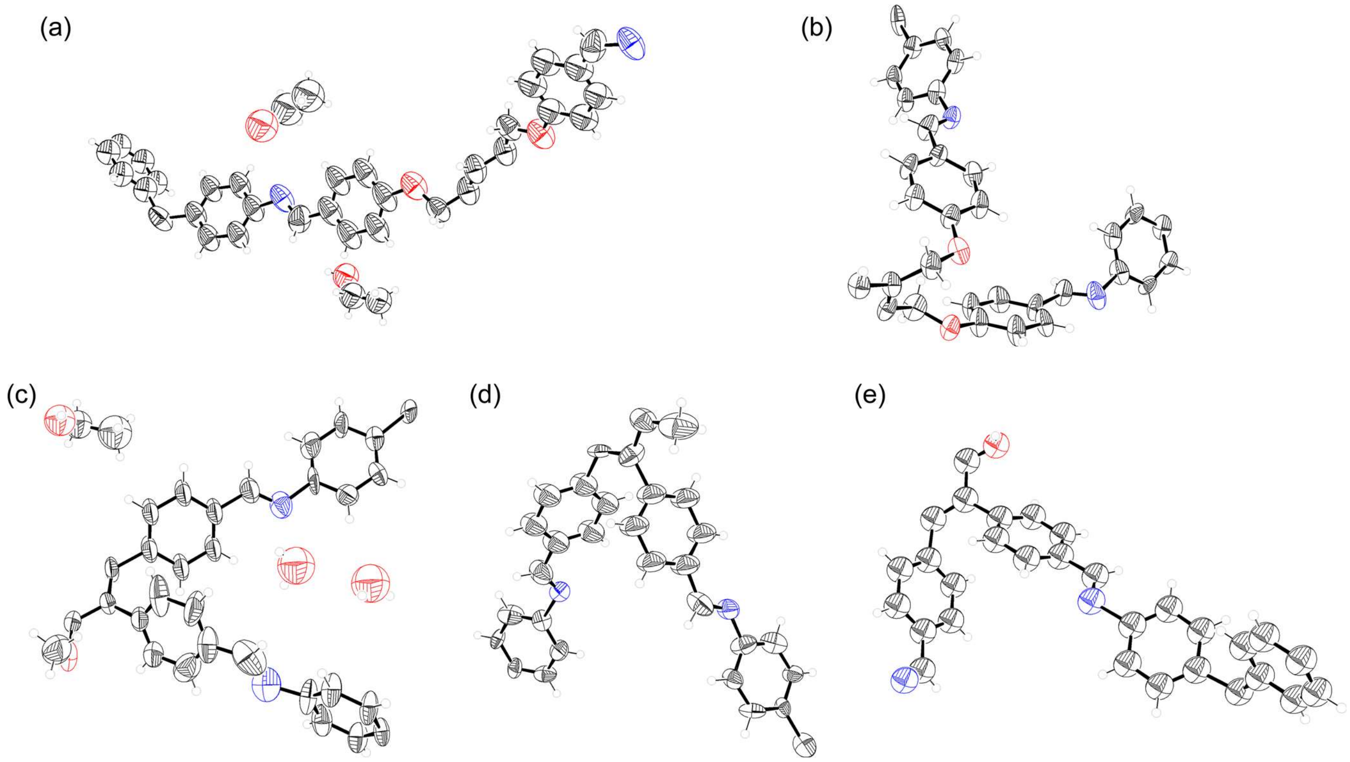

Supplementary Fig. 21 Ortep representation of 3D COFs with 50% probability ellipsoids. (a) E-FCOF-5 (CCDC number: 2115021), (b) C-FCOF-5 (CCDC number: 2115022), (c) 3D-TPB-COF-OMe (CCDC number: 2115023), (d) 3D-TPB-COF-Me (CCDC number:2115025), and (e) 3D-TPB-COF-OH (CCDC number: 2115024).

Supplementary Table 1 Summary of the cRED indexing and processing results for the E-FCOF-5 crystals.

Dataset	*a* (Å)	*b* (Å)	*c* (Å)	*α* (°)	*β* (°)	*γ* (°)	Volume (Å^3)	Rotation angle (°) / Data collection time (s)	Completeness (%)	Resolution (Å)	Redundancy
1#	14.58	8.51	26.49	90	92.97	90	3282.34	74.1/164.3	33	1.13	0.88
2#	14.58	8.61	26.23	90	91.49	90	3291.62	68.5/151.88	29	1.07	0.6
3#	14.33	8.42	26.42	90	91.57	90	3186.59	61.9/137.25	32	1.16	0.7
4	13.1	8.48	26.17	90	95.57	90	2893.43	61.9/137.25	33	1.16	0.69
5	13.39	8.78	23.85	90	104.9	90	2709.06	71.5/158.53	42	0.88	0.72
6#	13.45	7.94	25.66	90	93.79	90	2734.31	71.3/158.09	35	0.85	0.77
7	13.68	8.21	27.57	90	90.94	90	3096.04	75.9/168.29	35	1.15	0.92
8#	13.36	8.42	25.2	90	91.49	90	2833.81	34.3/76.05	27	1.29	0.49
9	13.87	8.7	26.11	90	90.79	90	3150.36	55.6/123.28	37	0.89	0.63
10#	13.73	8.66	26.05	90	90.17	90	3097.37	73.9/163.85	43	0.87	0.82
11#	13.52	8.67	25.93	90	89.95	90	3039.47	95.2/211.08	45	0.88	1.01
12#	13.6	8.66	26.19	90	90.54	90	3084.41	94.3/209.09	45	0.88	1.01
13#	13.51	8.67	26.04	90	90.37	90	3050.04	46.2/102.43	29	0.89	0.51
14#	13.52	8.63	25.99	90	90.51	90	3032.32	82.4/182.7	46	0.87	0.83
15	12.35	9.83	31.21	90	89.81	90	3788.88	57.8/128.15	33	0.89	0.42
16#	13.61	8.63	26.11	90	89.73	90	3066.69	78.2/173.39	40	0.91	0.88
17#	14.18	8.6	26.02	90	90.19	90	3173.07	50.5/111.97	28	0.89	0.56
18#	14.09	8.52	26.22	90	91.30	90	3146.81	84.1/186.47	46	0.88	1.04
19	13.45	9.41	28.61	90	80.52	90	3571.64	62.2/137.91	38	0.92	0.54
20#	13.95	8.53	26.02	90	90.65	90	3096.01	61.8/137.02	37	0.91	0.76
21#	13.96	8.6	26.16	90	92.41	90	3137.87	34.5/76.49	25.1	0.82	0.42
22#	13.8	8.46	26.74	90	90.99	90	3121.37	46.4/102.88	23	0.87	0.51

Datasets marked with # belong to the selected cluster for structure solution and refinement.

Supplementary Table 2 cRED experimental parameters, crystallographic data, and structure refinement details of E-FCOF-5.

**cRED experimental parameters and crystallographic data**	
Number of datasets	16
Tilt step	0.23°
Wavelength	0.0251 Å
Program for data process	*XDS*
Program for structure solution	*ShelxT*
Crystal system	Monoclinic
Unit cell dimensions	a = 13.710(3)Å<br>b = 8.634(1) Å<br>c = 26.409(5)Å<br>β = 91.409(5)°
Possible space group	*Pc, P*2/*c*
Resolution	0.82 Å
Completeness	96.5%
Redundancy	9.22
R_{int}	51.70%
No. of reflections	66388
No. of unique reflections	5879
**Structure refinement against cRED data**	
Formula	$2(C_{63} H_{46} N_4 O_4)$, $3(C_2 H_6 O)$
Crystal system	Monoclinic
Space group	*P*2/*c*
Unit cell dimensions	a = 13.710(3)Å<br>b = 8.634(1) Å<br>c = 26.409(5)Å<br>β = 91.409(5)°
Volume	3125.2(11)Å^3
Z	1
F(000)	506
Dataset (h, k, l)	-16~17, -10~10, -30~27
Tot., Uniq. Data, R_{int}	66388, 5879, 44.72%
Observed Data [Fo > 4sig(Fo)]	1788
$N_{\text{reflections}}$, $N_{\text{parameters}}$, $N_{\text{restraints}}$	5879, 134, 26
R_1, wR_2, *Gof*	0.3082, 0.6602,1.346
I/σ	3.7
ρ_{min} / ρ_{max} (e^- / Å^3)	-0.1/0.1

Supplementary Table S3 Summary of the cRED datasets indexing and processing results for the C-FCOF-5 crystals.

Dataset	*a* (Å)	*b* (Å)	*c* (Å)	*α* (°)	*β* (°)	*γ* (°)	Volume ($Å^3$)	Rotation angle (°) / Data collection time (s)	Completeness (%)	Resolution (Å)	Redundancy
1#	10.87	7.93	27.06	90	94.14	90	2326.45	51.9/115.07	31	0.89	0.79
2	10.86	8.42	27.54	90	88.48	90	2517.41	34.3/76.05	23	0.91	0.44
3#	11.25	7.92	27.98	90	96.01	90	2479.29	45.3/100.44	31	0.88	0.76
4#	10.62	7.75	27.16	90	95.63	90	2224.60	66.4/147.22	43	0.91	1.02
5#	11.43	7.94	26.59	90	94.63	90	2405.25	76.6/169.84	39	0.85	1.01
6#	11.00	7.59	26.56	90	94.19	90	2211.54	73.4/162.74	38	0.88	1
7#	11.43	7.48	26.51	90	93.07	90	2263.24	21.3/47.22	15	0.91	0.32
8#	11.71	7.88	27.22	90	98.00	90	2487.26	57/126.38	34	0.85	0.81
9	11.2	7.84	26.94	90	95.14	90	2356.03	41.1/91.13	25	0.86	0.7
10	10.24	7.89	27.78	90	94.80	90	2236.54	34.5/76.49	20	0.92	0.37
11#	11.02	7.57	27.09	90	97.46	90	2240.72	45.9/101.77	31	0.86	0.59
12	10.69	7.5	26.64	90	96.79	90	2120.86	45.6/101.10	22	0.84	0.6
13#	10.62	7.58	26.36	90	97.45	90	2104.05	40.9/90.68	23	0.87	0.54
14	10.92	7.88	26.35	90	95.15	90	2258.23	57.3/127.05	29	0.85	0.68
15	10.27	8.43	26.79	90	94.38	90	2312.58	47.2/104.65	29	0.82	0.42
16#	10.88	7.55	26.42	90	94.28	90	2164.17	81.1/179.82	46	0.87	1.04
17#	10.71	7.55	27.32	90	92.44	90	2207.10	41.6/92.23	25	0.85	0.57
18#	11.68	8.07	26.71	90	96.98	90	2498.96	64.7/143.45	38	0.86	0.96
19#	11.00	7.69	25.85	90	95.03	90	2178.20	57.6/127.71	33	0.9	0.81
20#	10.94	7.71	26.26	90	97.13	90	2197.80	34.3/76.05	24	0.92	0.53
21#	10.76	7.72	25.9	90	96.84	90	2136.11	45.4/100.66	32	0.79	0.65
22#	10.8	7.59	27.04	90	97.33	90	2198.38	45.6/101.10	29	0.88	0.71

Datasets marked with # belong to the selected cluster for structure solution and refinement.

Supplementary Table 4 cRED experimental parameters, crystallographic data, and structure refinement details of C-FCOF-5.

**cRED experimental parameters and crystallographic data**	
Number of datasets	16
Tilt step	0.23°
Wavelength	0.0251 Å
Program for data process	*XDS*
Program for structure solution	*ShelxT*
Crystal system	Monoclinic
Unit cell dimensions	a = 10.9511(2) Å<br>b = 7.7945(1) Å<br>c = 26.8214(6) Å<br>β = 95.247(3)°
Possible space group	*Pc, P*2/*c*
Resolution	0.81 Å
Completeness	94%
Redundancy	10.0
R_{int}	47.91%
No. of reflections	49801
No. of unique reflections	3973
**Structure refinement against cRED data**	
Formula	$C_{126}H_{92}N_8O_8$
Crystal system	Monoclinic
Space group	*P*2/c
Unit cell dimensions	a = 10.9511(2) Å<br>b = 7.7945(1) Å<br>c = 26.8214(6) Å<br>β = 95.247(3)°
Volume	2279.85(8) $Å^3$
Z	1
F(000)	506
Dataset (h, k,l)	-13~13, -8~8, -31~29
Tot., Uniq. Data, R_{int}	49801, 3973, 47.91%
Observed reflections [Fo > 4sig(Fo)]	2035
$N_{reflections}$, $N_{parameters}$, $N_{restraints}$	3973, 273, 0
R_1, wR_2, *Gof*	0.2192, 0.5377,1.437
I/σ	3.5
ρ_{min} / ρ_{max} (e^- / $Å^3$)	-0.3/0.2

Supplementary Table 5 Summary of the cRED datasets indexing and processing results for all the 3D-TPB-COF-OMe crystals.

Dataset	*a* (Å)	*b* (Å)	*c* (Å)	*α* (°)	*β* (°)	*γ* (°)	Volume ($Å^3$)	Rotation angle (°) / Data collection time (s)	Completeness (%)	Resolution (Å)	Redundancy
1	30.62	7.82	26.03	90	97.42	90	6180.55	53.36	37	0.83	0.67
2	31.54	7.62	26.13	90	96.11	90	6244.27	47.01	34.4	0.82	0.58
3	31.09	7.59	26.9	90	93.95	90	6332.52	87.50	54.7	0.79	0.95
4#	30.62	7.67	26.4	90	96.17	90	6164.24	103.25	65.9	0.87	1.55
5#	30.6	7.68	25.97	90	97.47	90	6051.23	103.39	63.9	0.88	1.62
6	31.04	7.65	25.94	90	96.78	90	6116.49	96.74	64.1	0.86	1.48
7	30.75	7.75	25.97	90	97.29	90	6138.89	10.56	10	0.87	0.14
8#	31.13	7.65	25.89	90	97.28	90	6115.73	19.26	17.1	0.87	0.28
9#	30.48	7.73	26.18	90	96.66	90	6126.60	71.30	57.8	0.87	1.06
10#	30.86	7.77	25.94	90	97.14	90	6171.63	92.52	60.2	0.88	1.49
11#	30.68	7.75	25.8	90	96.53	90	6094.65	37.57	32.6	0.89	0.61

Datasets marked with # belong to the selected cluster for structure solution and refinement.

Supplementary Table 6 cRED experimental parameters, crystallographic data, and structure refinement details of 3D-TPB-COF-OMe

**cRED experimental parameters and crystallographic data**		
Number of datasets	6	1
Tilt step	0.23°	0.23°
Wavelength	0.0251 Å	0.0251 Å
Program for data processing	*XDS*	*XDS*
Program for structure solution	ShelxT	ShelxT
Crystal system	Monoclinic	Monoclinic
Unit cell dimensions	a = 30.705(6) Å<br>b = 7.715(1) Å<br>c = 26.113(5)Å<br>β = 96.76(3)°	a = 30.705(6) Å<br>b = 7.715(1) Å<br>c = 26.113(5)Å<br>β = 96.76(3)°
Possible space group	*Cc, C*2/*c*	*Cc, C*2/*c*
Resolution	0.79 Å	0.86 Å
Completeness	91.8%	65.9%
Redundancy	8.43	1.55
R_{int}	41.36%	13.78%
No. of reflections	54798	7657
No. of unique reflections	5186	3562
**Structure refinement against cRED data**		
Formula	$C_{61}H_{42}N_4O_2$,<br>2($C_{1.08}H_{3.70}O_{0.54}$),<br>4(H2O)	$C_{61}H_{42}N_4O_2$
Crystal system	Monoclinic	Monoclinic
Space group	*C*2/*c*	*C*2/*c*
Unit cell dimensions	a = 30.705(6) Å<br>b = 7.715(1) Å<br>c = 26.113(5)Å<br>β = 96.76(3)°	a = 30.705(6) Å<br>b = 7.715(1) Å<br>c = 26.113(5)Å<br>β = 96.76(3)°
Volume	6143(2)Å^3	6143(2)Å^3
Z	4	4
F(000)	1037	1037
Dataset (*h*, *k*, *l*)	-36~36/-8~8/-30~28	-26~27/-8~8/-27~28
Tot., Uniq. Data, Rint	54798, 5186, 41.36%	7657,3562,13.78%
Observed reflections [*F*o > 4sig(*F*o)]	2001	705
$N_{reflections}$, $N_{parameters}$, $N_{restraints}$	5186,256, 16	3562,58, 17
R_1, wR_2, *Gof*	0.2280, 0.5606,1.356	0.2478, 0.5935,1.161
I/σ	4.8	4.5
ρ_{min} / ρ_{max} (e$^-$ / Å^3)	-0.2/0.2	-0.2/0.2

Supplementary Table 7 Summary of the cRED datasets indexing and processing results for all the 3D-TPB-COF-Me crystals.

**Dataset**	***a*** **(Å)**	***b*** **(Å)**	***c*** **(Å)**	***α*** **(°)**	***β*** **(°)**	***γ*** **(°)**	**Volume ($Å^3$)**	**Rotation angle (°) / Data collection time (s)**	**Completeness (%)**	**Resolution (Å)**	**Redundancy**
1	30.67	7.67	26.72	90	91.07	90	6284.48	120.7/267.62	69	0.90	1.32
2	30.76	7.62	26.89	90	90.61	90	6302.41	89.5/198.44	56	0.91	1.44
3#	30.98	7.64	26.77	90	90.67	90	6335.67	124.3/275.60	67	0.90	1.31
4#	31.01	7.72	26.49	90	90.26	90	6341.56	124.5/276.05	59	0.90	1.35
5#	30.98	7.67	26.81	90	90.14	90	6370.48	112.8/250.11	64	0.92	1.36
6#	30.95	7.69	26.37	90	89.82	90	6276.17	120.9/268.07	64	0.90	1.34
7#	30.95	7.65	26.55	90	89.85	90	6286.15	106.1/235.25	61	0.90	1.32
8#	31.38	7.62	26.13	90	90.06	90	6248.08	104.5/231.70	54	0.90	1.22

Datasets marked with # belong to the selected cluster for structure solution and refinement.

Supplementary Table 8 cRED experimental parameters, crystallographic data, and structure refinement details of 3D-TPB-COF-Me

**cRED experimental parameters and crystallographic data**	
Number of datasets	6
Tilt step	0.23°
Wavelength	0.0251 Å
Program for data processing	*XDS*
Program for structure solution	ShelxT
Crystal system	Monoclinic
Unit cell dimensions	*a* = 30.987(6) Å<br>*b* = 7.666(1)Å<br>*c* = 26.549(5)Å<br>*β* = 90.27(3)°
Possible space group	*Cc, C*2/*c*
Resolution	0.8 Å
Completeness	99.7%
Redundancy	8.65
R_{int}	38.88%
No. of reflections	67595
No. of unique reflections	6195
**Structure refinement against cRED data**	
Formula	C_{61} H_{42} N_4
Crystal system	Monoclinic
Space group	*C*2/*c*
Unit cell dimensions	*a* = 30.987(6) Å<br>*b* = 7.666(1)Å<br>*c* = 26.549(5)Å<br>*β* = 90.27(3)°
Volume	6307(2) $Å^3$
Z	4
F(000)	1024
Dataset (*h*, *k*, *l*)	-37~38/-9~9 -30~32
Tot., Uniq. Data, Rint	67595, 6195, 38.88%
Observed reflections [*F*o > 4sig(*F*o)]	2076
$N_{reflections}$, $N_{parameters}$, $N_{restraints}$	6195,187, 12
R_1, wR_2, *Gof*	0.2114, 0.5368,1.15
I/*σ*	4.2
ρ_{min} / ρ_{max} (e^- / $Å^3$)	-0.2/0.2

Supplementary Table 9 Summary of the cRED datasets indexing and processing results for the 3D-TPB-COF-OH crystals.

**Dataset**	***a*** **(Å)**	***b*** **(Å)**	***c*** **(Å)**	***α*** **(°)**	***β*** **(°)**	***γ*** **(°)**	**Volume ($Å^3$)**	**Rotation angle (°) / Data collection time (s)**	**Completeness (%)**	**Resolution (Å)**	**Redundancy**
1	29.52	7.81	25.97	90	92.976	90	5979.33	105.34	65.6	0.81	1.10
2#	28.82	8.01	26.02	90	95.857	90	5975.31	106.67	72.1	0.81	1.20
3#	29.08	7.94	26.36	90	95.855	90	6054.64	126.23	79.5	0.82	1.42
4#	28.97	7.96	26.11	90	96.798	90	5978.66	98.28	62.9	0.82	1.11
5#	29.45	7.83	27.28	90	95.948	90	6256.72	80.23	46.8	0.79	0.78

Datasets marked with # belong to the selected cluster for structure solution and refinement.

Supplementary Table 10 cRED experimental parameters, crystallographic data, and structure refinement details of 3D-TPB-COF-OH.

**cRED experimental parameters and crystallographic data**	
Number of datasets	4
Tilt step	0.23°
Wavelength	0.0251 Å
Program for data processing	*XDS*
Program for structure solution	*ShelxT*
Crystal system	Monoclinic
Unit cell dimensions	*a* = 29.550(6) Å<br>*b* = 7.842(1) Å<br>*c* = 26.730(5)Å<br>*β* = 96.86(3)°
Possible space group	*Cc, C*2/*c*
Resolution	0.8 Å
Completeness	95.5%
Redundancy	16.32
R_{int}	42.33%
No. of reflections	30380
No. of unique reflections	5517
**Structure refinement against cRED data**	
Formula	$C_{59} H_{38} N_4 O_2$
Crystal system	Monoclinic
Space group	C2/c
Unit cell dimensions	*a* = 29.550(6) Å<br>*b* = 7.842(1) Å<br>*c* = 26.730(5)Å<br>*β* = 96.86(3)°
Volume	6150(2)$Å^3$
Z	4
F(000)	1007
Dataset (*h*, *k*, *l*)	-34~32/ -9~9/ -33~30
Tot., Uniq. Data, R_{int}	30380, 5517, 42.33%
Observed reflections [*F*o > 4sig(*F*o)]	679
$N_{reflections}$, $N_{parameters}$, $N_{restraints}$	5117, 55, 22
R_1, wR_2, *Gof*	0.2603, 0.6391,0.863
I/σ	2.3
ρ_{min} / ρ_{max} (e^- / $Å^3$)	-0.2/0.2

Section 2: Reference

1. Liu, X., Li, J., Gui, B., Lin, G., Fu, Q., Yin, S., Liu, X., Sun, J. & Wang, C. *J. Am. Chem. Soc.* **143**, 2123-2129 (2021).
